# Clinical characteristics and short-term outcomes of hiv patients admitted to an african intensive care unit

**DOI:** 10.1186/2197-425X-3-S1-A351

**Published:** 2015-10-01

**Authors:** A Kwizera, J Nakibuuko, L Ssemogerere, E Ayebale, P Agaba, M Nabukenya, I Clarke

**Affiliations:** Makerere University College of Health Sciences, Anaesthesia and General Intensive Care, Kampala, Uganda; International Hospital Kampala, International Health Sciences University, Intensive Care Medicine, Kampala, Uganda; Mulago National Referral Hospital, Intensive Care Medicine, Kampala, Uganda; International Health Sciences University, Kampala, Uganda

## Introduction

Highly active antiretroviral therapy (HAART) for the treatment of the human immune deficiency virus (HIV) infection has been associated with improved survival among HIV infected patients world over even among patients admitted to the intensive care unit (ICU). There are no data regarding HIV patients admitted to low-income country ICUs. We sought to identify clinical characteristics and outcomes among HIV patients in a low-income country tertiary hospital.

## Objectives

We sought to identify ICU admission diagnoses and survival, to compare clinical characteristics and outcomes in patients according to survival, and to determine the predictors of survival. Our study results were compared to those of published studies in HIV infected patients admitted to the ICU in the era of HAART.

## Methods

We conducted a retrospective cohort study of all HIV infected patients admitted to the ICU of a university teaching hospital in Kampala, Uganda from 2009-2014. Medical records were reviewed. Clinical variables included CD 4 count, viral load, biochemical and haematological values. Serum albumin levels, need for vasopressors, mechanical ventilation, and acute physiological and chronic health evaluation (APACHE) score were documented. The primary outcome was survival to hospital discharge. Statistical significance was predetermined in reference to a p value of < 0.05.

## Results

There were 102 patients. Average age of patients was 38.4 and majority were females. Average length of stay was 4 days and mortality was 57%. The commonest admission diagnoses were Acute Respiratory Distress syndrome (ARDS), multi-organ failure, and sepsis. Average APACHE score was 24. Majority of patients were on HAART and most patients had a CD 4 count less than 100. Most patients were on septrin prophylaxis; mechanical ventilation and 36.6% were on vasopressors for septic shock. At multivariate analysis, APACHE II, mechanical ventilation and ARDS had a statistically significant association with mortality.

## Conclusions

ICU mortality of HIV patients is higher than that seen in higher income settings. ARDS is a leading reason for admission. ARDS, high APACHE II and the need for mechanical ventilation are significantly associated with mortality.
